# Association of Toll-like receptors 2, 3, and 4 genes polymorphisms with periapical pathosis risk

**DOI:** 10.4317/medoral.21081

**Published:** 2016-03-31

**Authors:** Ülkü Özan, Zeynep Ocak, Fatih Özan, Elif-Aybala Oktay, Orçun Toptaş, Halil Şahman, İhsan Yikilgan, Hasan Oruçoğlu, Kürşat Er

**Affiliations:** 1 Department of Endodontics, Faculty of Dentistry, Abant İzzet Baysal University, Bolu, Turkey; 2 Department of Medical Biology and Genetics, Faculty of Medicine, Abant İzzet Baysal University, Bolu, Turkey; 3 Department of Oral and Maxillofacial Surgery, Faculty of Dentistry, Abant İzzet Baysal University, Bolu, Turkey; 4 Department of Restorative Dentistry and Endodontics, GATA Dental Clinics, Ankara, Turkey; 5 Department of Oral and Maxillofacial Radiology, Faculty of Dentistry, Abant İzzet Baysal University, Bolu, Turkey; 6 Department of Restorative Dentistry and Endodontics, Faculty of Dentistry, Gazi University, Ankara, Turkey; 7 Department of Endodontics, Faculty of Dentistry, Akdeniz University, Antalya, Turkey

## Abstract

**Background:**

The aim of this study was to investigate the role of gene variations of Toll-like receptors (TLR) 2, 3, and 4 on genetic susceptibility to periapical pathosis.

**Material and Methods:**

One hundred patients were included in the study and divided into two groups as follows; Control Group (n=50) that have root canal treatment and no periapical lesion, Patient Group (n=50) that have root canal treatment and periapical lesion. TLR2 Arg753Gln, TLR3 (c.1377C/T) and TLR4 Asp299Gly and Thr399Ile polymorphisms were genotyped by using PCR-RFLP. Genotypical analysis of control and patient groups were investigated to disclose whether there is any association between periapical lesions and gene variations.

**Results:**

There are no significant statistical differences between control and patient groups according to TLR 2 and 4 gene sequence. On the contrary, CC allele detected 74% for TLR 3 in patient group, and this difference was found to be statistically significant (*p* < 0.005).

**Conclusions:**

According to these results, it can be suggested that patients with Toll-like receptor 3 gene polymorphisms could be susceptible to periapical pathosis.

**Key words:**Toll-like receptors, periapical pathosis, endodontics.

## Introduction

The persistent microbial infection within the root canal system of the affected tooth causes apical periodontitis which is an inflammatory disorder of periradicular tissues. Some problems result in persistent apical periodontitis such as inadequate aseptic control, improper access to cavity, missed canals, inadequate instrumentation, debridement, and leaking of restorations (1). However, it has recently been proposed that genetic predisposition in certain genes can contribute to persistent apical periodontitis (2).

The prime cause of periapical diseases is bacteria. Nonspecific inflammatory reactions occur when the bacteria or toxins invade the periapical region from an infected root canal. Then the process continues with specific inflammatory reactions that include the production of antibodies, complement, cytokines and an array of inflammatory mediators targeted at limiting the spread of infection and protecting the periapical tissues (3). In addition, a large and diverse community of viruses that have yet to be characterized in patients with periodontal disease live in the oral cavity (4). The double-stranded RNA (dsRNA) is a molecular pattern associated with viral infection and recognized by Toll-like receptor 3 (TLR3) (5).

Host defense in mammals copes with pathogens through 2 types of immunity: innate and adaptive immunity. Innate immunity functions as a pathogen sensor and contributes to the eradication of pathogens and the establishment of adaptive immunity. These functions heavily depend on pathogen recognition receptors (PRRs) (6). Among PRRs, a group of transmembrane proteins, Toll-like receptors (TLRs), are distinguished by their potent immuno adjuvant ability to activate antigen presenting cells (APCs) (7). TLRs are a family of receptors involved in the recognition of a wide range of microbial molecules, eg. lipopolysaccharide (LPS) from gram negative bacteria and peptidoglycan from gram positive bacteria, and innate and adaptive immune responses against invading pathogens (8). Binding of TLRs causes the production of inflammatory cytokines, including TNF-α and IL-12, and enhances the cells’ antimicrobial killing mechanisms and antigen-presenting capacity. Thirteen distinct TLR members have been identified in mammals. The endogenous ligands released from damaged tissues and necrotic cells, which are termed damage-associated molecular patterns (DAMPs), can also recognize and activate the TLRs (9). Numerous TLRs endogenous ligands have been identified. Most of them activate TLR2 and TLR4 (10). The mRNA released from necrotic cells may activate TLR3. TLR2 and TLR4 recognize bacterial cell-wall components, such as peptidoglycan (PGN) and LPS, respectively, whereas TLR3 recognizes the viral replicative intermediate double-stranded RNA (dsRNA) (8).

The activation of TLRs in the oral cavity might be a key event contributing to infectious exacerbations in oral cavity inflammatory disease (11). To our knowledge, there has been no genetic research into the relationship between TLRs 2, 3, and 4 and periapical pathosis. In this study, therefore, we aimed to detect whether there is a relationship between apical pathosis and the genetic variations of TLR2, 3 and 4.

## Material and Methods

- Sample population

Approval from the local ethics committee was obtained for the performance of this study. A total of 100 patients were included in the study. Patients were divided into 2 groups: control Group (CG) (n=50, 28 male and 22 female) that have root canal treatment and no periapical lesion; and Patient Group (PG) (n=50, 29 male and 21 female) that have root canal treatment and periapical lesion. TLRs 2, 3, and 4 gene variations were studied with peripheral blood samples obtained from patients. Inclusion criteria for patients were: to volunteer, to be an adult and to have had a root canal treatment. Exclusion criteria were: not to sign the informed consent form, to have uncontrolled diseases, to be pregnant, to be under chemotherapy and/or radiotherapy, and periodontally involved patients.

- Genomic DNA preparation

Two milliliters of whole blood samples was collected into ethylenediaminetetraacetic acid- anticoagulated tubes by the standard venipuncture method. Genomic DNA extraction from peripheral blood leucocytes was carried out using the salting-out method as described by Miller *et al.* (12).

- Polymerase chain reaction (PCR) and enzyme digest 

TLR2 Arg753Gln gene polymorphisms were analyzed by the method of Karaca *et al.* (13). The primers for TLR2 Arg753Gln were forward 5’-GCCTACTGGGTGGAGAACCT-3’ and reverse 5’-GGCCACTCCAGGTAGGTCTT-3’. PCR for TLR2 (Arg753Gln) was performed in a total volume of 25 µl containing approximately 100 ng DNA, 2.5 µl of 10X polymerase buffer, 2 mmol/L MgCl2, 0.2 mmol/L dNTPs, 0.4 µmol/l of each primer and 1 U of Taq polymerase (Thermo Scientific, Fisher Scientific-USA 300 Industry Drive, Pittsburgh, PA, USA) PCR program on T100 thermal cycler (Bio-Rad Laboratories 4000 Alfred Nobel Drive Hercules, CA, USA) thermal cycler was as follows: an initial denaturation step at 94 ºC for 4 min, followed by 33 cycles of 30 sec at 94 ºC, 30 sec at 56 ºC, 30 sec at 72 ºC, and a final extension step of 8 min at 72 ºC. The PCR products were digested by restriction endonuclease AciI (New England Biolabs,UK 75-77 Knowl Piece Wilbury Way Hitchin, UK) at 37 ºC overnight and then analyzed by 10% polyacrylamide gel electrophoresis. Bands of 228, 75 and 40 bp corresponded to TLR2 CC; 268, 228, 75, 40 bp bands were designated as heterozygous CG individuals; a band of 268,75 bp corresponded to the homozygous GG genotype.

PCR for TLR3 (c.1377C/T) was carried out in a total reaction volume of 25µLwith 20 pmol each of forward and reverse primer, (F) 5’CCAGG CATAAAAAGCAATATG and (R) 5’GGACCAAGGCAAAGGAGTTC, genomic DNA (200 ng), and PCR Master Mix (MBI Fermentas, Thermo Scientific). PCR conditions were as follows: initial denaturation of 95 ºC for 5 min, 35 cycles of 95 ºC for 45 s, 55 ºC for 45 s, and 72 ºC for 30 s, followed by a final extension of 72 ºC for 7 min. All these reactions were carried out in thermal cycler from T100 thermal cycler (Bio-Rad Laboratories 4000 Alfred Nobel Drive Hercules). PCR products were digested by restriction endonuclease TaqI (Thermo Scientific) at 65 ºC overnight and then analyzed by 10% polyacrylamide gel electrophoresis. Bands of 274 bp and 63 bp corresponded to TLR3 CC while 337, 274 and 63 bp bands were designated as heterozygous CT individuals; a band of 337 bp corresponded to the homozygous TT genotype.

Determination of the TLR4 gene polymorphisms was accomplished with PCR and restriction fragment length polymorphism by the method of Lorenz *et al.* (14). The primers for TLR4 Asp299Gly were forward 5’-GATTAGCATACTTAGACTACT ACCTCGA-3’ and reverse 5’-GATCAACTTCTGAAAAAGCATT CCCACC-3’. The primers for TLR4 Thr399Ile were forward 5’-GGTTGCTGTTCTCAAAGTGATTTTGGGACAA-3’ and reverse 5’-CCTGAAGACTGGAGAGTGAGTTAAATGCT-3’. Amplification conditions for TLR4 gene polymorphisms were described above for TLR2 polymorphisms, except for MgCl2 concentrations (4.0 mM MgCl2 for Asp299Gly and 3 mM for Thr399Ile). The cycling conditions comprised a hot start at 95º C for 10 min., followed by 35 amplification cycles at 95 ºC for 30 s, 62 ºC for 30 s (Asp299Gly) or 60 ºC (Thr399Ile) and 72 ºC for 25 s, followed by one elongation step at 72 ºC for 5 min. The digest reaction was set up using 4µL PCR product, appropriate restriction enzyme NcoI (TLR4 Asp299Gly) and HinfI (TLR4 Thr399Ile), 1 µL10 X enzyme buffer (Promega Corporation 2800 Woods Hollow Road Madison, WI, USA). It was incubated overnight at 37 ºC and electrophoresed in 10% polyacrylamide gel to identify the TLR4 alleles on the basis of the respective allele size. After digestion, the wild-type TLR4 allele sizes of 249 bp for the 299 residues and 406 bp for the 399 residues will not change; fragment sizes for carriers of the polymorphic allele will decrease to 23 bp for the 299 residues and 29 bp for the 399 residues. To confirm our PCR-RFLP results, PCR products for all genotypes were sequenced on an automated DNA sequencer (ABI 3130xl Genetic Analyzer; PE Applied Biosystems GenTech Scientific Inc., Arcade, NY, USA). The primer sequences and restriction enzymes used for PCR-RFLP methods for detecting each single nucleotide polymorphism (SNP) are shown in  Table 1.

**Table 1:**
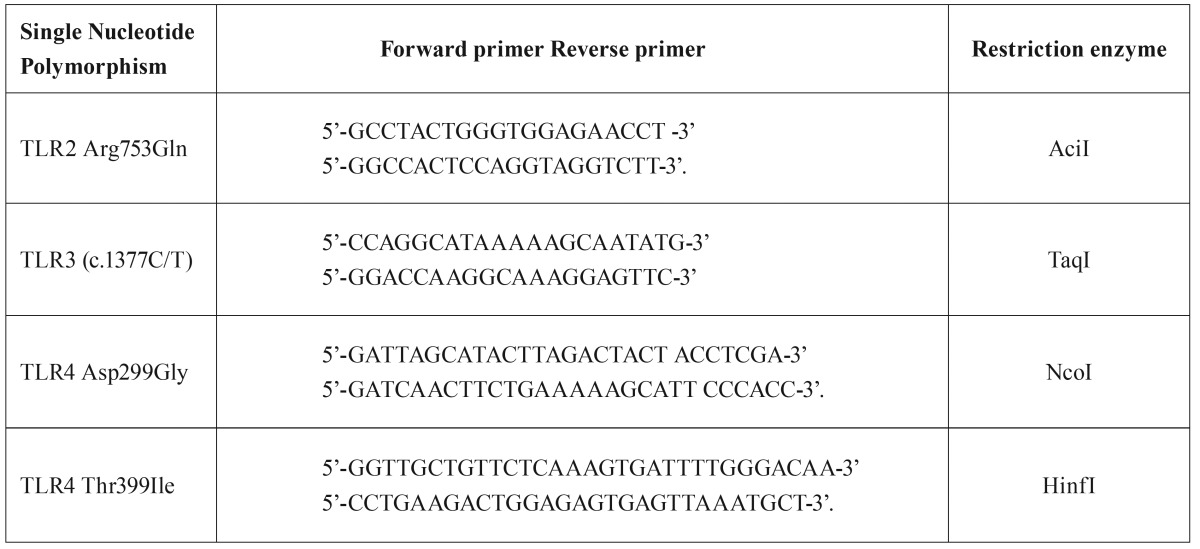
Primer sequences and restriction enzymes used for PCR-RFLP methods detecting the each single nucleotide polymorphism.

- Statistical Analysis

Analyses were performed with Epi Info version 3.5.3. Hardy-Weinberg equilibrium and the absence of LD were evaluated using the allele procedure of Epi Info version 3.5.3. All statistical tests were two-sided and a nominal *p* value low of 0.05 was considered statistically significant.

## Results 

A total of 50 patients with periapical pathosis and 50 age and sex-matched healthy controls were enrolled in the study. The mean age was 26.6±4.5 years. There were no significant differences between the two groups in terms of age and sex (*p* > 0.05 for both). Genotypic analyses of the patients were investigated to disclose whether there is any association between periapical lesions and gene variations. All of the 100 patients had CC genotype for TLR2 Arg753Gln and TLR4 Thr399Ile. Similar to the other groups all of the 100 patients had AA genotype for TLR4 Asp299Gly ( Table 2). In the patient group, we detected a TLR3 CC allele in 37 (74%) patients, CT allele in 9 (18%) patients and TT allele in 4 (8%) patients. In the control group, however, 19 (38%) subjects have a CC allele, 14 (28%) subjects have a CT allele and 17 (34%) subjects have a TT allele. There was a statistically significant difference between the groups in terms of CC allele frequency (*p * < 0.005).

**Table 2:**
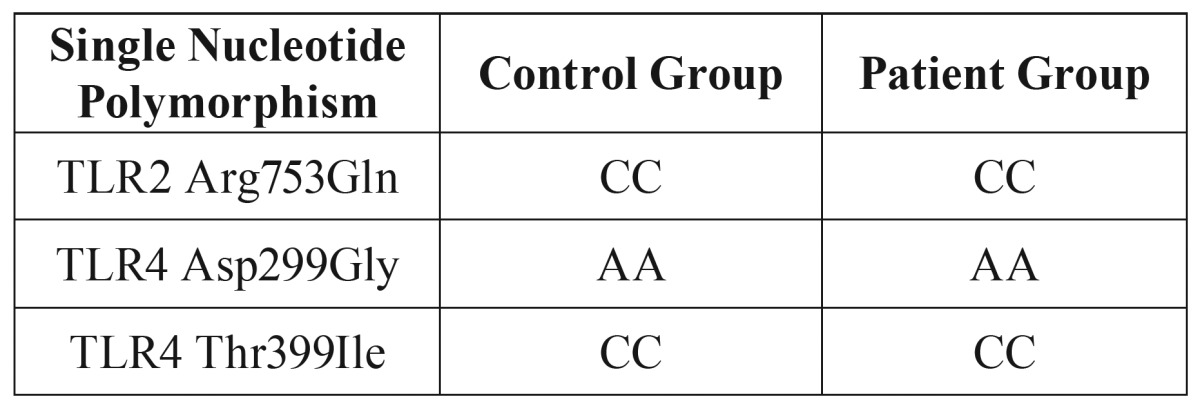
Genotypes of the control and patient groups except TLR 3.

## Discussion

Recent years have seen an exponential increase in the number of reports claiming links between genetic polymorphisms and a variety of medical diseases. Recently, endodontic research has contributed to this growth area. If we can understand the genetic basis of diseases, genetic tests to assess disease risk and to develop etiology-based treatments will soon be a reality (15).

The ultimate goals of root canal treatment are the complete healing of the periapical pathosis and the restoration of function. However, failure does occur despite all efforts and strict adherence to the principles of endodontic therapy in 14% to 16% of cases (16). Nair (17) defined apical periodontitis as a sequel to an endodontic infection that includes a dynamic encounter between microbial factors and host defenses. Periapical pathology is a multifactorial disease representing an interaction between a microbial challenge and immune response, which results in cytokine production and bone resorption.

There are limited numbers of clinical studies in the literature about the effect of genetic makeup on periapical diseases. Recently, the authors of this study investigated the expression of TLR2 in refractory periapical lesions (3). We found that periapical cysts are likely to be sustained by the immune system via reaction to bacterial antigens. In the present study, we investigated the role of gene variations of TLRs 2, 3 and 4 on genetic susceptibility to periapical lesions. In this study, we did not find any statistical relationship between patient and control groups at the TLR2 and TLR4 genes polymorphisms. The reason for this may be different epigenetic changes in gene activity that are not caused by changes in the DNA sequence.

TLR2 and TLR4 gene polymorphisms have previously been evaluated in some chronic inflammatory diseases (18). Ahmad-Nejad *et al.* (19) detected TLR2 Arg753Gln polymorphism in 11.5% of patients with atopic dermatitis. This polymorphism has been found to be associated with severe-to moderate atopic dermatitis together with the higher levels of total serum IgE and superantigen-specific IgE than the non-polymorphic atopic dermatitis. In addition, TLR2 Arg753Gln polymorphism carriers were found to have an increased risk for acne vulgaris in Chinese Han patients (20). TLR4 gene polymorphisms were found to be associated with Behcet’s disease, but not TLR2 Arg753Gln polymorphism (21). The reason for these studies’ results may be the different ethnic origin and persons that we selected for this study.

In this study, we have found the statistical difference between control and patient groups at the TLR3 gene polymorphisms. The findings of our study suggest that patients with TLR 3 gene polymorphisms could have a susceptibility to periapical pathosis. There is no established link between periapical pathogenesis and TLR3 gene polymorphisms; however, there is information about the links between different disease pathogeneses and those polymorphisms. In one of these studies, TLR 3 gene polymorphisms have been implicated in increased nasopharyngeal carcinoma risk. Lack of association of TLR 3 (c.1377C/T) gene polymorphism and increased risk for developing breast cancer has also been reported recently.

Several therapeutic agents targeting the TLRs are now under preclinical and clinical evaluation. However, TLRs act as double-edged swords because of their complexity. It is not clear that TLRs are either promoting or inhibiting disease progression. Furthermore, therapeutic agents targeting the TLRs must be able to antagonize the harmful effects resulting without affecting host defense functions. Nonetheless, the potential of harnessing and directing the innate immune system with drugs targeting TLRs for preventing or treating human inflammatory and autoimmune diseases as well as cancer appears to be promising (22).

Although, this study is the first report to describe a genetic marker that might identify people with an increased risk of periapical pathosis before the beginning of endodontic treatment, it has some limitations. The study subjects enrolled were all of similar ethnicity; since both periapical pathosis patients and, controls were of Turkish ethnicity, the possibility of population admixture was ruled out. The other limitation of this study is that we only focused on genotyped genes. Further studies are necessary to confirm our results in terms of gene expression and protein analysis of the interested protein by RNA experiments and western blotting in future.

Within the limitations of the present study, it may be concluded that patients with TLR 3 gene polymorphisms could have a susceptibility to periapical lesions.
